# Checklists for interpreting chest radiographs: a scoping review protocol

**DOI:** 10.1186/s13643-023-02327-w

**Published:** 2023-08-30

**Authors:** Khethiwe Margaret Sethole, Nombeko Mshunqane, Mable Kekana

**Affiliations:** 1 Department of Radiography, Faculty of Health Sciences, University of Pretoria, Hatfield, South Africa; 2 Department of Physiotherapy, Faculty of Health Sciences, University of Pretoria, Hatfield, South Africa

**Keywords:** Checklist, Chest interpretation, Medical doctors, Radiologists, Radiographers

## Abstract

**Introduction:**

What is known about checklists for interpreting chest radiographs? The question will guide the development of the inclusion criteria for the scoping review. Breaking down the scoping review question will allow the evaluation of inclusion and exclusion criteria for the protocol. The eligibility of the proposed research question will be assessed using the Population or Participants, Concept and Context (PCC) framework.

**Background:**

X-ray reporting can be standardised using checklists. Checklists may reduce the time needed to produce a comprehensive X-ray report and improve the quality and consistency of detecting abnormalities on chest radiographs. This scoping review aims to map the available literature on what is known about checklists for interpreting chest radiographs.

**Methods:**

We will follow the methodological framework for scoping reviews originally described by Arksey and O’Malley. The scoping review will include articles that describe checklists for reducing diagnostic errors, checklists for analysing chest radiographs, checklists for identifying abnormalities on chest radiographs and checklists for reporting chest radiographs in all settings. Search terms are chest radiographs, checklists, and chest X-rays. We will search for peer-reviewed articles and grey literature including dissertations and theses. We will search online databases including Ovid Medline and Ebscohost, to identify articles published in English from 1994 to 2022. The searched articles will undergo two levels of screening, first the title and abstract screening, then a full-text screening by two reviewers. Data from the selected articles will be extracted, using a tested extraction form and charted using the Joanna Briggs Institute guidelines.

**Results:**

The results will be collated, summarised and discussed including any limitations of the included articles. The articles will be summarised in a table, as well as narratively. The distribution of studies will be summarised quantitatively and the numerical analysis will provide an overview and identify knowledge gaps. Content analysis will map different checklists available for chest interpretation.

**Discussion:**

The results of the scoping review will be used to develop a checklist that will be used by medical doctors in collaboration with radiographers working in settings where there are no radiologists on-site, for interpreting chest radiographs.

**Systematic review registration:**

Scoping review protocol registered with Open Science Framework on 27 July 2022. Registration https://doi.org/10.17605/OSF.IO/JS5PQ

**Supplementary Information:**

The online version contains supplementary material available at 10.1186/s13643-023-02327-w.

## Introduction

The South African health system is facing a quadruple burden of diseases. Compounding this is the great disparity between the available healthcare professionals and the workload burden they face. One way to alleviate the burden of shortage of radiologists, is the interprofessional collaboration involving regular interaction between professionals, which values the expertise and contributions that various healthcare professionals bring to patient care. An inter-professional practice-based intervention tool can be deployed in the workplace.

The review is done to explore, map and summarise the extent and nature of published research on checklists available to interpret chest radiographs. The intent is to develop a checklist, to standardise chest interpretation amongst radiographers and medical doctors working in resource-constrained settings, where there are no radiologists on site. The checklist will reduce omission errors caused by poor inter-professional communication between radiographers and GPs, inadequate image evaluation and inadequate searching for abnormal patterns, during chest interpretations. The checklist will enhance interprofessional communication, reduce omission errors, reduce interpretation times, delays, inter-reader variability and excessive radiologist’s workload.

## Background

Chest X-rays are the most common radiological examination performed in hospitals. Medical doctors use chest X-rays as a first-line diagnostic tool in the initial screening, diagnosis, monitoring and predicting outcomes of many diseases. Medical doctors working in district hospitals refer patients for X-rays and are mandated to analyse, and interpret the images giving a diagnosis because there are no radiologists on site. Medical doctors, on several occasions, have approached radiographers to help interpret radiographic images. Diagnostic radiographers are professionals who perform radiographic examinations, integrating patient history with clinical data and imaging techniques to obtain quality diagnostic images.

Radiologists are medical practitioners who undergo intensive postgraduate education and training to become experts in analysing, interpreting and detecting abnormalities on radiographic images giving a diagnostic report. Currently, there is a universal shortage of radiologists to handle the changes brought about by technological advances in digital imaging modalities like magnetic resonance imaging (MRI), computed tomography (CT) scans and interventional radiology.

Shortages of radiologists may lead to poor X-ray reporting turnaround times. For example, in 2015 there were only an estimated 650 registered radiologists in South Africa, equating to 1.2 radiologists per 100,000 people in a population of 52 million [1]. These staff shortages led to general radiographic images not being reported, or reporting being delayed well beyond internationally acceptable reporting time parameters [2]. Lack of reporting of radiographic images by radiologists may contribute to misdiagnosis or mismanagement of patients [2]. Globally, radiographer-led reporting and image interpretation were introduced to improve patient management, reduce waiting times and costs, enhance patient safety and have been recommended as a solution to bolster the radiology workforce [3, 4].

In South Africa, the radiographer code of practice states that: Radiographers who perform the radiographic examination may only provide a voluntary, verbal opinion to the referrer [5]. Due to the radiographer’s code of practice, other solutions need to be explored to assist in the interpretation of radiographic images, where radiographers and medical doctors collaborate to accurately interpret images of the chest [6]. Such inter-professional collaboration (IPC) can improve patient care [7]. Effective IPC can be facilitated by implementing formal communication tools, inter-professional meetings and checklists [8]. According to the Royal College of Radiologists [9], checklists are a catalyst to improve communication, support teamwork and improve patient safety. Hospital imaging protocols should specify IPC processes to standardise service delivery ensuring the consistency in practice and flow of information.

Effective IPC is vital in resource-poor settings that experience staff shortages. In South Africa, there are two healthcare sectors, the public sector which provides healthcare to approximately 75% of the population, and the private sector, with most patients having medical insurance. Hospitals in the public sector, including district hospitals, cater to South Africans in rural and urban areas, including mainly unemployed and low-income earners. Hospitals in the public sector have lower human-resourcing ratios, financial constraints and ageing infrastructure [10]. There are 240 district hospitals in South Africa, all of which have X-ray facilities but do not have radiologists on site. In these settings, IPC can be facilitated if there are adequate tools to promote communication. Effective use of available resources in a resource-constrained environment, such as the South African healthcare system, is a more cost-effective alternative.

Checklists are tools that can standardise reporting, produce comprehensive reports in shorter times and improve the detection of abnormalities on chest radiographs. Checklists reduce errors of omission, summarise large quantities of information and allow the formulation of reliable evaluations [11]. Checklists are recognised as a potential tool for preventing diagnostic errors caused by a lack of IPC [12]. This protocol proposes a review of the literature on existing checklists for analysing chest radiographs. To the best of our knowledge, no reviews have been conducted on this topic. The majority of reviewed studies focused on determining the accuracy of radiographers’ ability to interpret radiographic images of the skeleton and the chest. One study explored and described the reporting experiences of radiographers and medical practitioners. What is known from a systematic review conducted is that SA diagnostic radiographers have the basic knowledge required to contribute significantly to the clinical environment in interpreting images.

Currently, there is a universal shortage of radiologists and few available works in tertiary and private hospitals. Some cases are sent to tertiary hospitals for reporting and the report will only be available after two to three days. On several occasions, medical doctors have approached Radiographers to help interpret radiographic images, but both radiographers and medical doctors are not trained to detect abnormalities in radiographic images. To alleviate the challenge of delayed reporting, inter-professional collaboration (IPC) between radiographers and medical doctors can improve patient care. Effective IPC can be facilitated by implementing formal communication tools, inter-professional meetings and checklists. Checklists are a catalyst to improve communication and support teamwork.

It is unclear what kind of information is available in the literature about checklists to interpret chest radiographs and what kind of support is available for the collaboration of radiographers and medical doctors in terms of the interpretation of chest radiographs. For these reasons, a scoping review will be conducted to systematically map the research done in this area, as well as to identify any existing gaps in knowledge. This proposed scoping review aims to, map and summarise the extent, range and nature of published research on existing checklists used for interpreting chest radiographs. The results of the scoping review will be used to develop a checklist that will be used by medical doctors and radiographers working in settings where there are no radiologists on-site, as a collaborative knowledge-sharing strategy to enhance the interpretation of chest radiographs.

## Methods

Scoping reviews are useful for mapping key concepts on a topic, identifying available literature, and identifying gaps in existing research. We will follow the methodological framework for scoping reviews originally described by Arksey and O’Malley [13] and further refined by the Joanna Briggs Institute [14]. This framework comprises five stages: (1) identify the research question by clearly identifying the purpose of the review, (2) identify the relevant studies using a three-step literature search to balance breadth and comprehensiveness, (3) select studies using a team approach, (4) chart the data in a tabular and narrative format and (5) collate the results to identify the implications of the study [14]. We will follow the Systematic Reviews and Meta-Analyses extension for Scoping Reviews (PRISMA-ScR) checklist to report the flow of information [15]. We will register the protocol in the Open Science Framework and will publish the protocol in a peer-reviewed journal to prevent unnecessary duplication. Due to the iterative nature of a scoping review, methodology changes to the protocol may occur. We will report any changes to the protocol.

An objective is a clear, succinct statement that conveys why the review should be conducted, what the review will add to the reader's knowledge in the field and what specifically is being investigated.

The objectives of this scoping review are to:

1. Explore the research conducted on checklists used for interpreting chest radiographs including publication dates, volumes, yearly distributions, proportions and geographical location.
2. Explore research methods and designs used to develop checklists for interpreting chest radiographs including purpose, context, study population, sample size, design and methods of data collection.

### Scoping review question

The scoping review question for this study is: What is known about checklists for interpreting chest radiographs? The question will guide the development of the inclusion criteria for the scoping review. Breaking down the scoping review question allows the evaluation of inclusion and exclusion criteria for the protocol. The eligibility of the proposed research question will be assessed using the Population or Participants, Concept and Context (PCC) framework depicted in Table 1 [14]. The PCC framework guided the construction of a clear and meaningful title.

**Table 1:** PCC framework for defining the research question for this scoping review on the available checklists for analysing chest radiographs

**Population: chest radiographs**	Chest radiographs, chest X-rays, radiography of lungs or thoracic cage
**Concept: checklists**	Checklists for structured chest interpretations Checklists for inter-professional communication Checklist to reduce radiographic omission errors Checklists to identify abnormal patterns on chest radiographs Checklists to evaluate and analyse radiographs
**Context: international**	Literature from all government and private health settings on chest image or radiograph interpretation

### Inclusion criteria

We will include peer-reviewed articles that focus on checklists to enhance IPC between radiographers and medical doctors, articles that describe checklists for reducing diagnostic errors, checklists for analysing chest radiographs, checklists for identifying abnormalities on chest radiographs and checklists for reporting chest radiographs in all settings. We will include all peer-reviewed articles and grey literature, including theses and dissertations included in relevant databases. We will also snowball sample the reference lists of relevant articles. All peer-reviewed articles will have an abstract and clearly stated aim. Only articles in English, published between 1994 and 2022 will be included in the review. We chose the start date of 1994 because it covered important policy changes in South Africa.

### Search strategy or design

Using the PCC framework, we will search online databases using the appropriate indexing terminology and Medical Subject Headings (MeSH) terms. A librarian will design and refine our search strategy.

The conduction of a preliminary research will take place in two databases, namely Ovid Medline and Ebscohost, which are appropriate for searching literature on chest image interpretation. The preliminary search will be conducted using the following terms: chest radiographs, checklist and chest X-rays. Next, we will analyse the titles, abstracts, and index terms of retrieved articles. New terms will be added and the search strategy refined. The Boolean operators “AND” and “OR” will be used as needed. The second search will be conducted and all the results will be imported into a reference management software. Additional studies will be identified after searching all references cited in the included studies. A statement will be included about the reviewers’ intent to contact authors of primary studies or reviews for further information, if necessary.

### Study selection

**Fig. 1:**
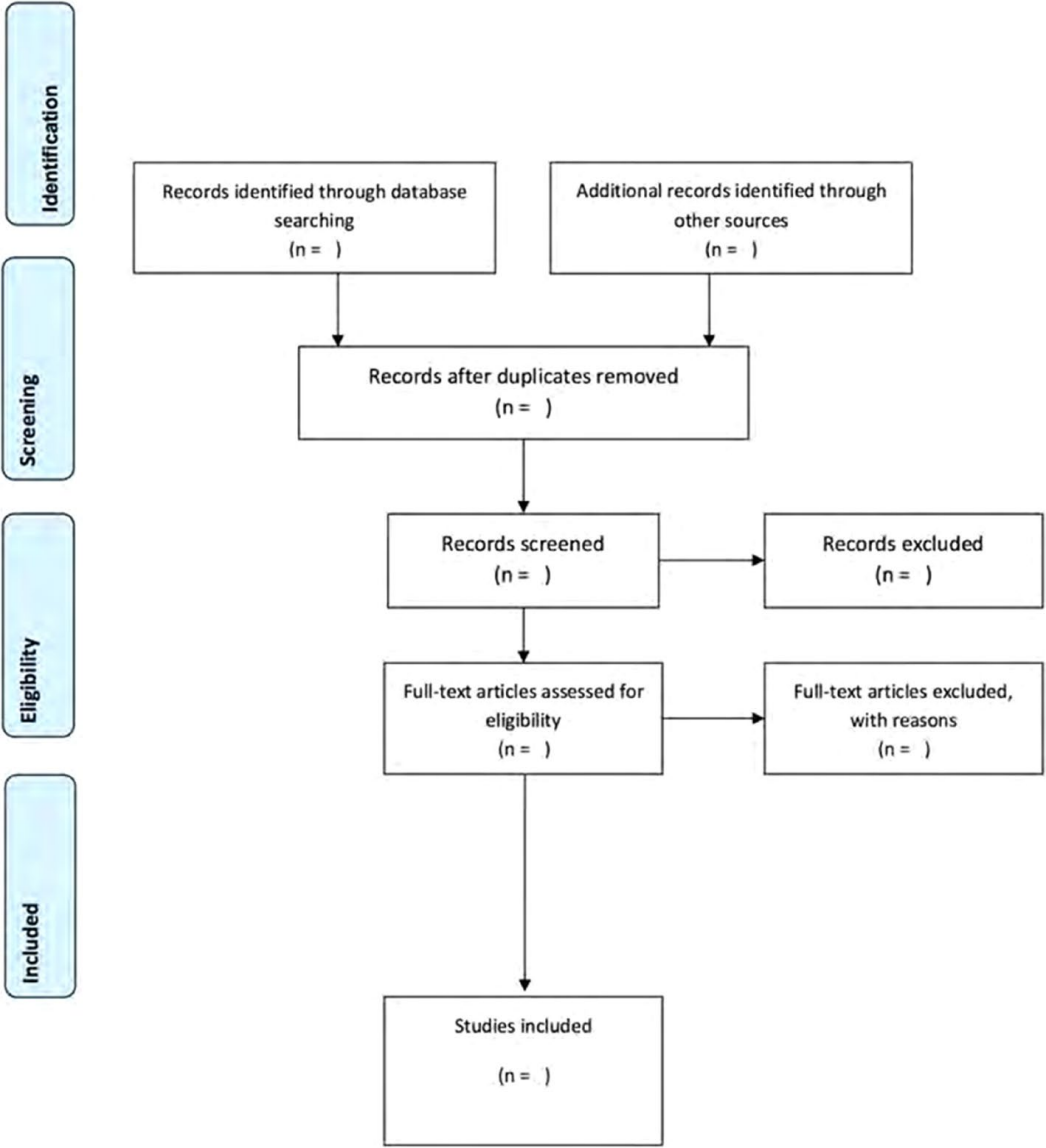
PRISMA flow diagram showing the process followed to identify relevant articles on checklists for analysing chest radiographs [15]. The flowchart details the review decision process, indicating the results from the search, removal of duplicate citations, study selection, full retrieval and additions from a third search, and the final summary presentation. The “Results” section will identify how many studies were identified and selected

Two levels of screening will take place for the identification of relevant literature. Firstly, two independent reviewers will screen the titles and abstracts of all articles. Articles that do not concur with the PCC framework will be excluded. In the second step, the two reviewers will independently assess the full-text articles to determine whether they meet the inclusion criteria. Any disagreements will be resolved by a third investigator until a full consensus is obtained. Scoping reviews do not exclude articles according to methodological quality, thus the methodological quality of the included studies will not be evaluated. The categorisation of data will be held via the process outlined in the PRISMA flow diagram (Fig. 1).

### Extracting and charting data

The data will be extracted using the proposed tool shown in Table 2, which includes the fields suggested by the Joanna Briggs Institute [14], and will be summarised descriptively according to the objectives and questions of the scoping review. The proposed data extraction tool will be compiled in Excel spreadsheets. Before the data extraction, the data extraction tool will be tested using five studies to determine agreement within the research team. The data extraction tool will be modified based on the reviewers’ feedback and it will constantly be updated throughout the scoping review. Two reviewers will then independently read and extract data from each article.

**Table 2:** Proposed data extraction tool that will be used to chart data from all included articles. These fields are suggested by Joanna Briggs Institute

Field	Data
Author(s):	
Year of publication:	
Country:	
Aims/purpose:	
Study population:	
Sample size:	
Methodology:	
Intervention type and comparator:	
Duration of the intervention:	
Outcomes’ measures:	
Key findings:	

## Results

The results will be collated, summarised and discussed in detail, including any limitations of the included articles. The included articles will be summarised in a table, as well as narratively. The extent, nature, and distribution of studies will be summarised quantitatively. This simple numerical analysis will provide an overview and identify knowledge gaps. Secondly, content analysis will take place for the mapping of different available checklists.

## Discussion

To our knowledge, this will be the first scoping review of literature on checklists used to interpret chest radiographs. The scoping review will provide an overview of the current evidence on checklists for interpreting chest radiographs and will identify in which areas systematic reviews or primary research are needed. This review is strengthened by the use of transparent and reproducible procedures. The protocol of the present scoping review describes in detail, the population, concept and context, data sources, search strategy, data extraction, and analysis which will be used. Limitation of the review will be due to the search being limited to certain databases, from 1994 to 2022 and only English articles considered.

## Conclusion

A scoping review is done to identify types of checklists available to interpret chest radiographs, with the aim of developing a checklist that can be used by medical doctors in collaboration with radiographers working in areas where there are no radiologists on-site to standardise the interpretation of chest radiographs.

### Supplementary Information

**Additional file 1.**

## Data Availability

Not applicable as this is a protocol for a scoping review. The search strategy has been discussed.

## Abbreviations

CT: Computerised tomography
EQUATOR: Enhancing the QUAlity and Transparency Of health Research
IPC: Inter-professional collaboration
JBI: Joanna Briggs Institute
MRI: Magnetic resonance imaging
Mesh: Medical Subject Headings
PACS: Picture archiving and communication system
PPC: Population (or Participants), Concept, and Context
PRISMA-ScR: Preferred Reporting Items for Systematic reviews and Meta-Analyses extension for Scoping Reviews
SA: South Africa
