# Supplementary material for: Checklists for interpreting chest radiographs: a scoping review protocol
Source: Syst Rev. 2023 Aug 30;12:152. doi: 10.1186/s13643-023-02327-w (PMC10466731; doi:10.1186/s13643-023-02327-w)
Supplement: Supplementary file 1 — Additional file 1. [file 13643_2023_2327_MOESM1_ESM.docx]

**Preferred Reporting Items for Systematic reviews and Meta-Analyses extension for Scoping Reviews (PRISMA-ScR) Checklist**

| **SECTION** | **ITEM** | **PRISMA-ScR CHECKLIST ITEM** | **REPORTED ON PAGE #** |
| --- | --- | --- | --- |
| **TITLE** | | | |
| Title | 1 | Checklists for interpreting chest radiographs: A scoping review protocol. | FRONT PAGE |
| **ABSTRACT** | | | |
| Structured summary | 2 | X-ray reporting can be standardised using a checklist.  Aim: The scoping review aims to map the available literature on what is known about checklists for interpreting chest radiographs.  Method: The scoping review will include articles that describe checklists for reducing diagnostic errors, checklists for analysing chest radiographs, checklists for identifying abnormalities on chest radiographs and checklists for reporting chest radiographs in all settings. Search terms are chest radiographs, checklists, and chest X-rays. We will search for peer-reviewed articles and grey literature including dissertations and theses. We will search online databases including Ovid Medline and Ebscohost, to identify articles published in English from 1994 to 2022. The searched articles will undergo two levels of screening, first the title and abstract screening, then a full-text screening by two reviewers  Data from the selected articles will be extracted, using a tested extraction form and charted using the Joanna Briggs Institute guidelines. The distribution of studies will be summarised quantitatively and the numerical analysis will provide an overview and identify knowledge gaps. Content analysis will map different checklists available for chest interpretation.  Results: The results will be collated, summarised and discussed including any limitations of the included articles. | Page1 |
| **INTRODUCTION** | | | |
| Rationale | 3 | The review will be done to explore, map and summarize the extent and nature of published research on checklists available to interpret chest radiographs. The intent is to develop a checklist, to standardise chest interpretation amongst radiographers and medical doctors working in resource-constrained settings, where there are no radiologists on site. | Page2-3 |
| Objectives | 4 | The question for this study is: What is known about checklists for interpreting chest radiographs?  The objectives are to:   1. Explore the research conducted on checklists used for interpreting chest radiographs 2. Explore research methods and designs used to develop checklists for interpreting chest radiographs including purpose, context, study population, sample size, design and methods of data collection. | Page 5 |
| **METHODS** | | | |
| Protocol and registration | 5 | Protocol registered with Open science framework on 27 July 2022. Registration https://doi.org/10.17605/OSF.IO/JS5PQ; | Page 5 |
| Eligibility criteria | 6 | Include peer-reviewed articles that focus on checklists to enhance communication between radiographers and medical doctors, articles that describe checklists for reducing diagnostic errors, for analysing chest radiographs, for identifying abnormalities on chest radiographs and for reporting chest radiographs in all settings. Include grey literature, theses and dissertations included in relevant databases. Snowball sample the reference lists of relevant articles. All peer-reviewed articles will have an abstract and clearly stated aim. Only articles in English, published between 1994 and 2022 will be included in the review. Start date of 1994 because it covered important policy changes in South Africa. | Page 6 |
| Information sources* | 7 | Using the PCC framework, we will search online databases using the appropriate indexing terminology and Medical Subject Headings (MeSH) terms. The conduction of a preliminary research will take place in two databases, namely Ovid Medline and Ebscohost. Other databases will be included in the second search. | Page7 |
| Search | 8 | The preliminary search will be conducted using the terms: chest radiographs, checklist and chest X-rays. The Boolean operators ‘AND’ and ‘OR’ will be used as needed. The second search will be conducted and all the results will be imported into a reference management software. Additional studies will be identified after searching all references cited in the included studies. | Page7 |
| Selection of sources of evidence† | 9 | Two levels of screening will take place for the identification of relevant literature. Firstly, two independent reviewers will screen the titles and abstracts of all articles. Articles that do not concur with the PCC framework will be excluded. In the second step, the two reviewers will independently assess the full-text articles to determine whether they meet the inclusion criteria. | Page 8 |
| Data charting process‡ | 10 | Charting data will be from a form that will be tested by the team before the use, and will be done independently in duplicate. | Page 8 |
| Data items | 11 | Not applicable | n/a |
| Critical appraisal of individual sources of evidence§ | 12 | Not applicable | n/a |
| Synthesis of results | 13 | Not applicable | n/a |
| **RESULTS** | | | |
| Selection of sources of evidence | 14 | Not applicable | n/a |
| Characteristics of sources of evidence | 15 | Not applicable | n/a |
| Critical appraisal within sources of evidence | 16 | Not applicable | n/a |
| Results of individual sources of evidence | 17 | Not applicable | n/a |
| Synthesis of results | 18 | Not applicable | n/a |
| **DISCUSSION** | | | |
| Summary of evidence | 19 | Not applicable | n/a |
| Limitations | 20 | Not applicable | n/a |
| Conclusions | 21 | Not applicable | n/a |
| **FUNDING** | | | |
| Funding | 22 | No funding | Page10 |

JBI = Joanna Briggs Institute; PRISMA-ScR = Preferred Reporting Items for Systematic reviews and Meta-Analyses extension for Scoping Reviews.

* Where *sources of evidence* (see second footnote) are compiled from, such as bibliographic databases, social media platforms, and Web sites.

† A more inclusive/heterogeneous term used to account for the different types of evidence or data sources (e.g., quantitative and/or qualitative research, expert opinion, and policy documents) that may be eligible in a scoping review as opposed to only studies. This is not to be confused with *information sources* (see first footnote).

‡ The frameworks by Arksey and O’Malley (6) and Levac and colleagues (7) and the JBI guidance (4, 5) refer to the process of data extraction in a scoping review as data charting*.*

§ The process of systematically examining research evidence to assess its validity, results, and relevance before using it to inform a decision. This term is used for items 12 and 19 instead of "risk of bias" (which is more applicable to systematic reviews of interventions) to include and acknowledge the various sources of evidence that may be used in a scoping review (e.g., quantitative and/or qualitative research, expert opinion, and policy document).

*From:* Tricco AC, Lillie E, Zarin W, O'Brien KK, Colquhoun H, Levac D, et al. PRISMA Extension for Scoping Reviews (PRISMAScR): Checklist and Explanation. Ann Intern Med. 2018;169:467–473. [doi: 10.7326/M18-0850](http://annals.org/aim/fullarticle/2700389/prisma-extension-scoping-reviews-prisma-scr-checklist-explanation).
